# [Corrigendum] Omi/HtrA2 pro-apoptotic marker differs in various hepatocellular carcinoma cell lines owing to ped/pea-15 expression level

**DOI:** 10.3892/or.2024.8804

**Published:** 2024-08-28

**Authors:** Zongquan Xu, Yu Chen, Guohui Xu, Cheng Peng, Enyu Liu, Yunguang Li, Jun Niu, Changhai Li

Oncol Rep 33: 905–912, 2015; DOI: 10.3892/or.2014.3656

Subsequently to the publication of the above paper, an interested reader drew to the authors' attention that the control western blots shown for Fig. 1A and B on p. 908 and Fig. 8A and C on p. 911 were apparently the same, where different experiments were intended to have been portrayed.

After having re-examined their original data files, the authors realized that these figures had been published with the control western blots shown incorrectly for Fig. 1A and 8C. The corrected versions of this pair of figures are shown on the next page. Note that the corrections made to these figures do not affect the overall conclusions reported in the paper. The authors are grateful to the Editor of *Oncology Reports* for allowing them the opportunity to publish this Corrigendum, and apologize to the readership for any inconvenience caused.

## Figures and Tables

**Figure 1.**
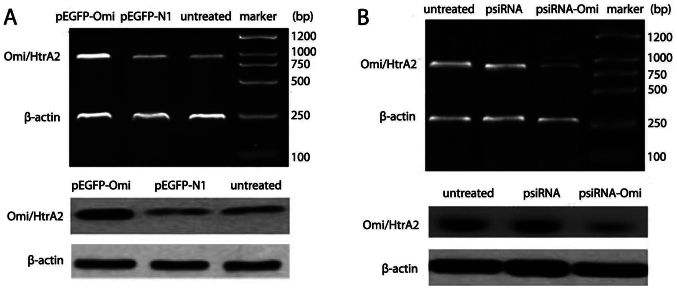
Omi/HtrA2 mRNA and protein expression in HepG2 transfected with Omi/HtrA2 expression vector pEGFP-Omi or Omi/HtrA2 small interference RNA expression vector psiRNA-Omi

**Figure 8.**
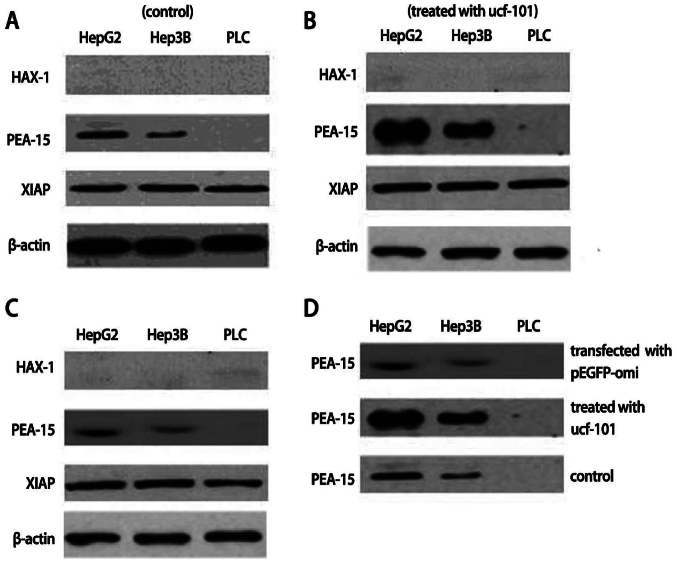
Protein expression of XIAP, ped/pea-15 (PEA-15) and HAX-1 in HepG2, Hep3B and PLC cells. XIAP overexpressed in HepG2, Hep3B and PLC cells and its expression had no difference among them and HepG2, Hep3B and PLC cells had no HAX-1 expression. (A) PLC cells were devoid of ped/pea-15 expression although ped/pea-15 was overexpressed in HepG2 and Hep3B cells. However, ped/pea-15 expression was higher in HepG2 cells than that in Hep3B cells. (B) The protein expression of XIAP and HAX-1 in HepG2, Hep3B and PLC cells showed no change, whereas ped/pea-15 protein expression increased in HepG2 and Hep3B cells following cell exposure to ucf-101. (C) The protein expression of XIAP and HAX-1 in HepG2, Hep3B and PLC cells did not change, whereas the ped/pea-15 protein expression decreased in HepG2 and Hep3B cells when cells were transfected into pEGFP-Omi. (D) The change of ped/pea-15 protein expression in HepG2 cells was more evident than that in Hep3B cells when transfected into pEGFP-Omi or exposed to ucf-101.

